# Supplementary material for: Asymmetric Synthesis of Quaternary Hydantoins via a Palladium-Catalyzed Aza-Heck Cyclization
Source: J Am Chem Soc. 2025 Nov 14;147(49):44692–8. doi: 10.1021/jacs.5c16022 (PMC12703750; doi:10.1021/jacs.5c16022)

D:\data\2024\DWatson_Idowu_TDI01073 02/12/24 17:05:58

DWatson_Idowu_TDI01073 #34-56 RT: 0.24-0.35 AV: 23 NL: 1.20E10
T: FTMS + p ESI Full ms [133.5000-2000.0000]

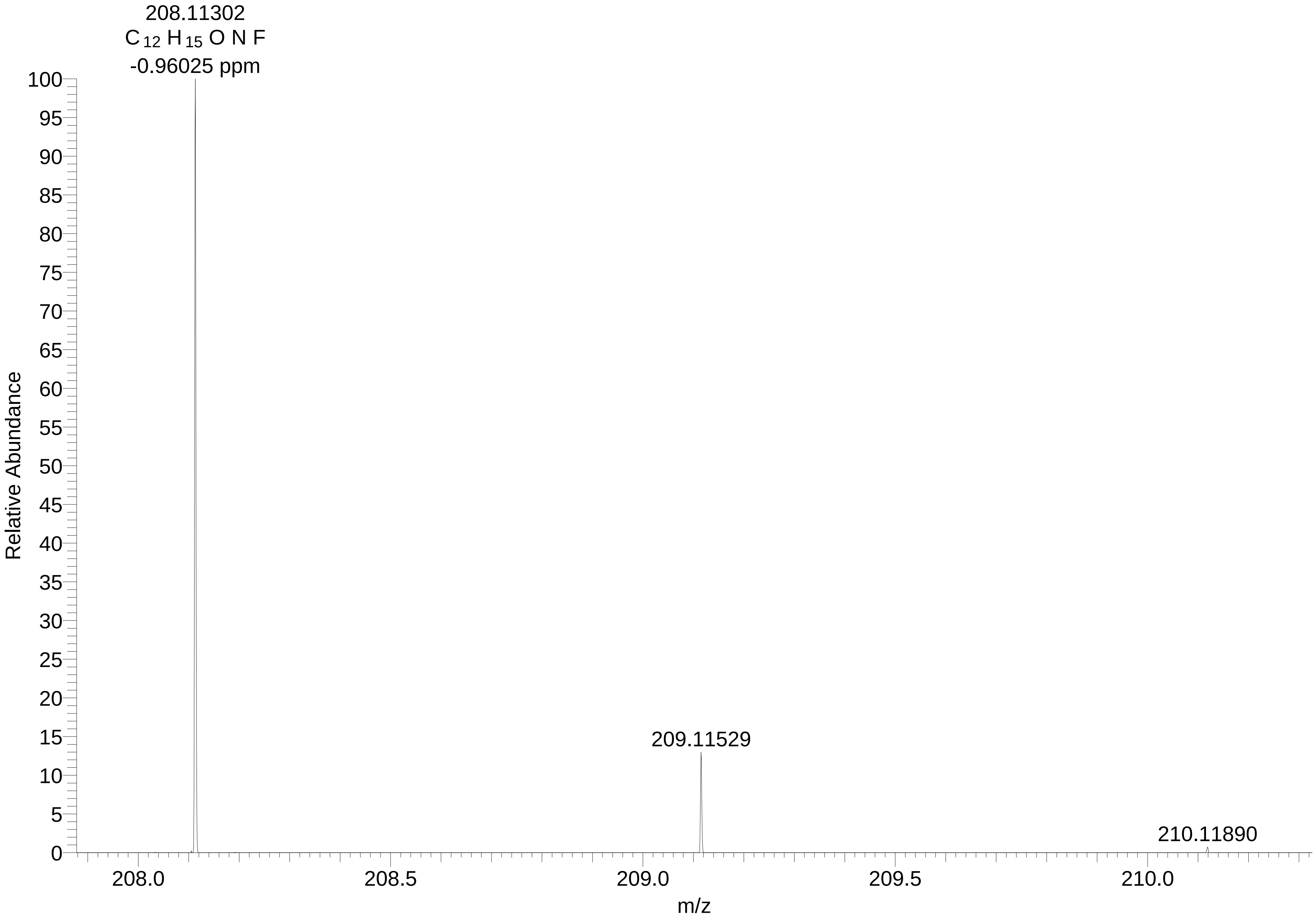

Supplement: Supplementary file 2 [file ja5c16022_si_002.zip › All NMR FID Files/S15/S15_AllNMR/HRMS/TDI01073.pdf]
